# Integrated miRNA-Seq and mRNA-Seq Study to Identify miRNAs Associated With Alzheimer’s Disease Using Post-mortem Brain Tissue Samples

**DOI:** 10.3389/fnins.2021.620899

**Published:** 2021-03-23

**Authors:** Qingqin S. Li, Diana Cai

**Affiliations:** Neuroscience, Janssen Research & Development, LLC, Titusville, NJ, United States

**Keywords:** hsa-miR-132, hsa-miR-212, differentially express genes (DEGs), mRNA-seq, miRNA-seq, Alzheimer’s disease

## Abstract

Alzheimer’s disease (AD), the leading form of dementia, is associated with abnormal tau and β-amyloid accumulation in the brain. We conducted a miRNA-seq study to identify miRNAs associated with AD in the post-mortem brain from the inferior frontal gyrus (IFG, *n* = 69) and superior temporal gyrus (STG, *n* = 81). Four and 64 miRNAs were differentially expressed (adjusted *p*-value < 0.05) in AD compared to cognitively normal controls in the IFG and STG, respectively. We observed down-regulation of several miRNAs that have previously been implicated in AD, including hsa-miR-212-5p and hsa-miR-132-5p, in AD samples across both brain regions, and up-regulation of hsa-miR-146a-5p, hsa-miR-501-3p, hsa-miR-34a-5p, and hsa-miR-454-3p in the STG. The differentially expressed miRNAs were previously implicated in the formation of amyloid-β plaques, the dysregulation of tau, and inflammation. We have also observed differential expressions for dozens of other miRNAs in the STG, including hsa-miR-4446-3p, that have not been described previously. Putative targets of these miRNAs (adjusted *p*-value < 0.1) were found to be involved in Wnt signaling pathway, MAPK family signaling cascades, sphingosine 1-phosphate (S1P) pathway, adaptive immune system, innate immune system, and neurogenesis. Our results support the finding of dysregulated miRNAs previously implicated in AD and propose additional miRNAs that appear to be dysregulated in AD for experimental follow-up.

## Introduction

Alzheimer’s Disease (AD), a neurodegenerative brain disease and the leading cause of dementia, is characterized by symptoms such as memory loss, depression, disorientation and confusion (Caraci et al., 2010; Jahn, 2013; Klimova et al., 2015; Lanctôt et al., 2017). In 2019, over 50 million people were estimated to live with dementia worldwide, and this number is projected to increase to more than 152 million by 2050, as populations age (Alzheimer’s disease international, 2019). The total worldwide cost of dementia is estimated to be more than $1 trillion, and this figure is predicted to increase to $2 trillion by 2030 (Alzheimer’s disease international, 2019). In addition, there is increased risk for emotional distress and negative mental and physical health outcomes for caregivers (Weller and Budson, 2018; Reed et al., 2020).

Two pathological hallmarks of AD are amyloid plaques and neurofibril tangles (NFT). Despite international drug development efforts in the past decade, there are only five approved therapeutics available for AD, including three cholinesterase inhibitors (donepezil, galantamine, and rivastigmine), one *N*-methyl-D-aspartate (NMDA) receptor antagonist (memantine), and a donepezil/memantine combination therapy (Cummings et al., 2018). With the advent of new technologies, AD research has expanded to explore epigenetic control mechanisms such as histone modification, DNA methylation/hydroxy-methylation and non-coding RNA-associated gene silencing (Fischer, 2014). Greater understanding in these areas may enable earlier detection of AD, which can help reduce the burden of the disease, and expand possible therapeutic targets against AD.

MicroRNAs (miRNA), ∼18–25 nucleotides in length, encompass the largest group of small non-coding RNAs (Bartel, 2009; Kosik, 2010). They primarily regulate expression at the post-transcriptional level via recognition of specific binding sites located in the 3′-untranslated region (UTR) of their target messenger RNAs (mRNAs), which leads to the degradation of these mRNAs and translational repression (Bartel, 2009; Roshan et al., 2009; Kosik, 2010; Bartel, 2018). One miRNA can regulate multiple mRNAs (Bartel, 2004), suggesting that dysregulation of a single miRNA can have a dramatic downstream effect.

Previous studies suggest miRNAs targeting amyloid precursor protein (*APP*) or *BACE1* expression affect AD pathogenic pathways and alter the risk and/or progression of the disease (Hébert et al., 2008; Wang et al., 2008; Boissonneault et al., 2009; De Smaele et al., 2010). miR-107 and miR-29b target the β-site APP cleaving enzyme BACE1, whose proteolytic activity generates amyloid β polypeptide, the primary component of amyloid plaques found in the brains of AD patients (Blennow et al., 2006; Hébert et al., 2008; Nelson and Wang, 2010; O’Brien and Wong, 2011; Zhang et al., 2011; Takahashi et al., 2017).

Neurofibril tangles are another pathological hallmark of AD and involve the accumulation of an abnormally phosphorylated form of the protein tau within neurons (Tolnay, and Probst., 1999; Avila, 2006). The miRNA, miR-132, has been implicated in tau metabolism as it regulates exon splicing of tau and modulates neuronal tau phosphorylation (Hébert et al., 2012). Additionally, in the last decades, many publications have implicated miRNA in the role of AD disease progression, particularly in 3′-UTR dysregulation of targeted mRNAs of *APP*, *PSEN1*, *PSEN2*, and *APOE4* genes (Avila, 2006; Vilardo et al., 2010; Dickson et al., 2013; Liu, Song et al., 2014; Santa-Maria et al., 2015; Reddy et al., 2016).

While several miRNAs have been implicated in AD and have been proposed as potential biomarkers for AD, there has been high variability between the reported data, including lack of methodological standards, heterogeneity across samples of patients with AD, and studies of small sample size (Moradifard et al., 2018; Herrera-Espejo et al., 2019; Nagaraj et al., 2019). Takousis et al. (2019) conducted a comprehensive meta-analysis on 147 independent datasets across 107 publications in which they quantitatively assessed 461 meta-analyses across brain, blood and CSF specimens. From their meta-analysis, 25, 5, and 32 miRNAs showed study wide significant differential expression in brain, CSF and blood-derived specimens, respectively (Takousis et al., 2019). Of those, 5 miRNAs were significant in both blood and brain. This study highlights the largest number of replicable miRNAs implicated in AD across sample types. Disease-associated miRNAs represent a new class of targets for the development of miRNA-based therapeutic modalities using antimir oligonucleotides (Stenvang et al., 2012).

In this study, we undertook a hypothesis free approach using miRNA-Seq and aimed to identify additional novel miRNAs and validate reported miRNAs associated with AD using post-mortem brain samples. In mild dementia, transcriptional changes have been shown to occur with greater frequency in the superior temporal gyrus (STG). As the dementia progresses from mild to moderate and severe, transcriptional changes in the STG are even greater than normal and occur across greater regions in the brain, including the inferior frontal gyrus (IFG) (Haroutunian et al., 2009). To obtain representation across different brain regions and different disease states, we obtained samples from the IFG and STG regions of subjects with AD, mild cognitive impairment (MCI) or cognitively normal controls. We confirmed differentially expressed miRNAs previously reported for AD patients and identified additional miRNAs to be pursued in future studies.

## Materials and Methods

### Cohort

Post-mortem tissue samples from the IFG and STG brain regions of subjects with AD or MCI or cognitively normal controls were acquired from Banner Sun Health Research Institute (Beach et al., 2008, 2015). These brain samples came from subjects who were volunteers in the Arizona Study of Aging and Neurodegenerative Disorders (AZSAND) and the Brain and Body Donation Program, a longitudinal clinicopathological study of healthy aging, cognition, and movement in the elderly since 1996 in Sun City, Arizona? All subjects signed an Institutional Review Board-approved informed consent, allowing both clinical assessments during life and several options for brain and bodily organ donation after death. Patient brain samples with short post-mortem intervals (PMI), large numbers of slices located in the frontal and temporal cortex (by Brodmann’s area) were prioritized to ensure the homogeneity of samples for group-wise comparison in the study. Overlapping samples from the same cohort was also described in a recent epigenome-wide association study (EWAS) and a mRNA-Seq study (Li et al., 2020; Li and De Muynck, 2021).

### miRNA-Seq Data Generation

As described previously (Li et al., 2020), genomic DNA and total RNA, including miRNA were simultaneously purified from brain tissue samples using the AllPrep DNA/RNA/miRNA Universal Kit (QIAGEN Inc., Germantown, MD, United States) following the manufacturer’s guidelines. Approximately 20–30 mg of tissue was used for each extraction. RNA quantity and quality assessment was performed according to established laboratory procedures, where RNA quantity was assessed by NanoDrop 2000 spectrophotometers (Thermo Scientific, Waltham, MA United States), and RNA mass and integrity was assessed using Agilent 2100 Bioanalyzer system and the Agilent RNA 6000 Nano Kit (Agilent Technologies, Santa Clara, CA, United States) at the site of RNA extraction and again at sequencing facility. A total of 181 RNA samples with RNA integrity number (RIN) greater than 5.8 proceeded to the library construction step for miRNA-Seq data generation.

RNA sample QC was performed with an Agilent 2100 Bioanalyzer to identify samples total mass and integrity. 1 mg of RNA from each sample was processed by following the protocol of the NEBNext Small RNA Library Prep Set for Illumina^®^. Briefly, small RNA fragments of size 18–40 nt were selected by PAGE gel. 3′ adaptors were added, followed with 5′ adaptor ligation. First strand was generated by reverse transcription PCR. PCR amplification was followed to enrich the cDNA fragments. The PCR products were purified with PAGE gel.

The final library was quantitated by Agilent 2100 Bioanalyzer instrument and real-time qPCR. The qualified libraries were amplified on cBot to generate the clusters on the flow cell, and sequenced at single end with read length of 50 bp on HiSeq 4,000 platform yielding ∼29 million reads (average) per sample.

### mRNA-Seq Data Generation

The mRNA-Seq study was reported previously (Li et al., 2020; Li and De Muynck, 2021). STG RNA samples (*n*_*case*_ = 24, *n*_*control*_ = 38) from the same cohort above with RNA integrity number (RIN) greater than 6 were proceeded to the library construction step for mRNA-Seq data generation. Libraries were constructed using TruSeq Stranded mRNA Library Prep (Illumina Inc., San Diego, CA, United States) according to manufacturer’s protocol using 200 ng of input RNA and sequenced using HiSeq4000 (Illumina Inc., San Diego, CA, United States) using paired end (100 bp × 2) sequencing to a sequencing depth of 40 M reads (or 8 G data). All data generation was conducted by laboratory personnel blinded as to the clinical phenotype. This dataset was used as a look up data set for evidence of differential gene expression (nominal *p*-value < 0.05) in the opposite direction among the predicted or experimentally observed targets of differentially expressed miRNA (FDR adjusted *p*-value less than 0.1).

### Data Pre-processing

miRNA-Seq data were evaluated using mirnaQC (Aparicio-Puerta et al., 2020) for benchmarking purpose, and FastQC (v0.11.5). mirnaQC provides several quality features to help researchers identify issues in samples. These features are provided as absolute values and ranked with a percentile calculated from a corpus of more than 36,000 samples. As expected, mean miRNA length was 22, and in average 50, 2.2, 25.6, 0.7, 0.1, 1.1, and 1.1% of the reads were miRNA, rRNA, tRNA, mRNA, antisense mRNA, snRNA, and snoRNA, respectively, while 8.4% of the reads were unassigned. For average Phred score, raw number of reads, percentage of reads in analysis, this study samples ranked in top quartile (best performance) among the 36,000 reference samples. The alignment/qualification, however, is not the data moving forward for the data analysis. For downstream data analysis, the adapters were trimmed using SOAPnuke (v1.5.0) (Chen et al., 2018) and sequence reads were aligned using bowtie (v1.1.1) against reference genome hg38 (Langmead et al., 2009). We used “-v 1 -m 10 -a –best –strata” options for the bowtie alignment step to report only those alignments in the best alignment “stratum,” where the alignments in the best stratum are those having the least number of mismatches with no more than one mismatch. Transcript quantification was performed using featureCounts (Liao et al., 2014) from subread (v2.0.0) package using default options (which do not count multi-mapping reads at all) against all 2,652 miRNA genes in miRbase (Kozomara and Griffiths-Jones, 2014) (v22.1). The combination of bowtie options and featureCounts options used in the analysis was essentially identical to using “–best –strata -k 1 -m 1” for bowtie which was demonstrated to be maximizing reads correctly mapped while minimizing reads incorrectly mapped to hairpin and non-hairpin loci using both simulated and real data (Ziemann et al., 2016). miRNA count data was transformed to log2-counts per million (logCPM) using voom function in R package limma (v3.38.3) (Ritchie et al., 2015), while estimating the mean-variance relationship to compute appropriate observation-level weights for downstream linear modeling. Surrogate variables are covariates constructed directly from high-dimensional data that could be used in subsequent analyses to adjust for unknown, unmodeled, or latent sources of noise (Leek and Storey, 2007, 2008). We used R package sva (v3.30.1) (Leek et al., 2012, 2019) to detect and estimate surrogate variables for unknown sources of variation to remove artifacts in the high-throughput experiments. Removing batch effects and using surrogate variables in differential expression analysis have been shown to reduce dependence, stabilize error rate estimates, and improve reproducibility (Leek et al., 2010). Excluding 1 duplicated sample, 2 samples from the superior frontal gyrus (SFG), 6 samples with discrepant phenotype between sources (each frozen tissue sample was labeled with the brain region, clinical diagnosis status, and the linked donor identifier and therefore a consistency check could be performed between the clinical diagnosis status on the sample label vs. the linked phenotype associated with the donor identifier on the sample label), as well as 22 additional samples from the middle temporal gyrus (MTG), results from a total of 150 samples used in the downstream analyses are reported in this study.

mRNA-Seq data were processed using cutadapt (v1.13) (Martin, 2011), STAR (v2.5.3a) (Dobin et al., 2013). Transcript quantification was performed using RSEM (v1.3.0) (Li and Dewey, 2011) against all 26,000 genes in NCBI RefSeq database (version date; 2015-07-17). Similar to the miRNA data analysis, we used the sva package (Leek et al., 2012) to detect hidden nuisance factors in the mRNA-Seq dataset.

### Identification of Differentially Expressed miRNA and mRNA

Both miRNA and mRNA differential gene expression analyses were performed using linear regression models via R package limma (Ritchie et al., 2015). The primary analysis was to identify differentially expressed miRNAs and mRNAs between clinical diagnosis groups. Due to the small sample size for the MCI group, the primary contrast was between AD cases and cognitively normal controls, while MCI samples were only used in graphical display. The mRNA analysis results were used as a look up dataset for putative miRNA targets of interest. A secondary analysis was to identify miRNAs correlated with Braak neurofibrillary stages (I–VI) (Braak and Braak, 1991), used as a quantitative endpoint. For each end point of interest (either diagnosis status or Braak stage), the statistical models corrected for the top two or five surrogate variables, gender, and age. We also accounted for the correlation between multiple brain regions from the same donor using the duplicateCorrelation function from limma.

### Over-Representation Analysis (ORA) of Putative Targets of Differentially Expressed miRNAs

Ingenuity Knowledge Base (QIAGEN, Redwood City, CA, United States) is a database with curated and integrated miRNA targets from various sources and literature [miRecords (Xiao et al., 2009), TarBase (Karagkouni et al., 2018), TargetScan (Agarwal et al., 2015), and Ingenuity Expert Findings] and classified the targets into three groups: experimentally observed, predicted with high confidence [cumulative weighted context score (CWCS) less than −0.4 for TargetScan v7.2], and predicted with moderate confidence (CWCS between −0.2 and −0.4 for TargetScan v7.2) (Supplementary Text 1). To minimize the false positive prediction, we require that miRNA differential expression FDR less than 0.1 and the direction of miRNA dysregulation has to be in opposite direction compared the target of miRNA, and the corresponding mRNA shall have a differential gene expression *p*-value less than 0.05.

ORA (Boyle et al., 2004) was performed using https://www.gsea-msigdb.org/gsea/msigdb/compute_overlaps.jsp which has a broader background gene set assumption and test over-representation at higher levels of the ontology hierarchy. Gene ontology databases used included c2.cp and c5 subsets of Molecular signatures database (MSigDB) (Liberzon et al., 2011).

### Cross Reference of miRNA Findings From This Study With Reported Meta-Analysis and Literature Findings

Results from a recent meta-analysis study on brain, CSF, and blood miRNAs (Takousis et al., 2019) served as the primary source for systematic comparison to the findings from this study. Overlapping miRNA findings was reported.

### mRNA-miRNA Correlation Analysis

In order to identify potential targets of miRNAs, paired miRNA-mRNA correlation analysis was performed using R package Hmisc v4.2.0, which only examined the correlation between miRNA and the predicted target from TargetScan databases (v7.2 and v6.2 conserved sites only with context score less than −0.2). We used the fsva function in the sva R package to perform frozen surrogate variable analysis (Parker et al., 2014) to remove nuisance batch effects from both miRNA and mRNA datasets and used the adjusted version of datasets for correlation analysis.

## Results

### Sample Characteristics

The age at death, gender, post-mortem interval (PMI), and *APOE* genotype and additional clinical characteristics are summarized in Table 1. The clinical diagnosis status was used as the case status phenotype for this study. This is consistent with the Consortium to Establish a Registry for Alzheimer’s Disease (CERAD) score (Mirra et al., 1991), a semiquantitative measure of neuritic plaques used to classify patients. The case status was also consistent with the National Institute on Aging and the Reagan Institute (NIA-Regan) AD criteria (Hyman and Trojanowski, 1997). Both AD and cognitively normal control clinical diagnostic groups have subjects in Braak neurofibrillary stages III–IV.

**TABLE 1:** Demographic and clinical characteristics of the samples used in the miRNA-seq assay.

**Brain region**	**STG**			**IFG**		
**Clinical diagnosis**	**Cognitively normal**	**Mild cognitive impairment**	**Alzheimer’s disease**	**Cognitively normal**	**Mild cognitive impairment**	**Alzheimer’s disease**
**Sample size**	***N* = 39**	***N* = 15**	***N* = 27**	***N* = 24**	***N* = 9**	***N* = 36**
Age at death, Mean (*SD*)	80.15 (7.25)	84.60 (5.51)	86.37 (6.37)	80.42 (7.32)	83.67 (6.38)	86.19 (7.25)
Gender, Female *n* (%)	12 (30.8)	7 (46.7)	12 (44.4)	6 (25.0)	4 (44.4)	24 (66.7)
PMI, Mean (*SD*)	3.26 (2.40)	3.22 (0.91)	3.37 (2.30)	3.08 (1.74)	3.22 (0.92)	3.03 (1.91)
**NIA-Reagan criteria (Hyman and Trojanowski, 1997), *n***						
Criteria not met	38	13		24	8	
Not AD	1	1			1	
Low			1			1
Intermediate		1	12			19
High			14			16
**Semiquantitative measure of neuritic plaques CERAD score (Mirra et al., 1991), *n***						
Criteria not met	5	3		2	1	
Not AD	24	5		14	4	
Possible AD	10	7		8	4	1
Probable AD			8			7
Definite AD			19			28
**Braak stage**						
I	9	2		15	1	
II	9	1	1	9	2	1
III	14	2	3	4	1	4
IV	7	9	9	6	5	15
V			10			9
VI		1	4			7
*APOE* genotype*, *n*						
e2/e2				1		
e2/e3	6	4	3	3	2	3
e3/e3	23	7	10	14	4	19
e3/e4	9	4	10	6	3	10
e2/e4	1		3			
e4/e4						3

*SD, standard deviation; STG, superior temporal gyrus (BA22); IFG, inferior frontal gyrus (BA44); PMI, post-mortem interval; CERAD (Mirra et al., 1991), Consortium to Establish a Registry for Alzheimer’s Disease*.

*1 STG and 1 IFG sample, respectively, has missing APOE genotype.

### Identification of Differentially Expressed miRNA Genes (DEGs)

**FIGURE 1:**
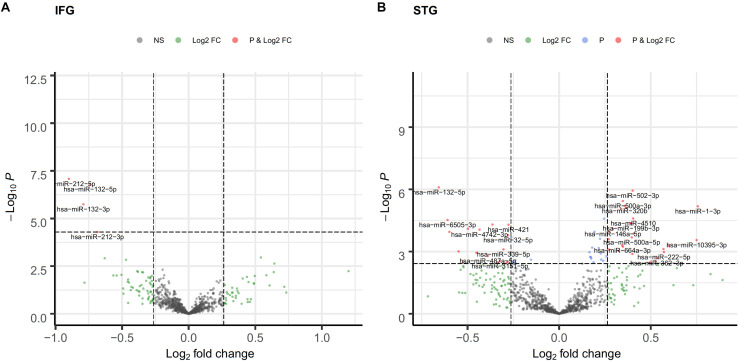
Volcano plot of differentially expressed miRNA in the IFG **(A)** and STG **(B)**. log2(fold change) is plotted against -log10(*p*-value), where *p*-value is from differential miRNA expression test. The vertical dash line represents fold change of 1.2, while the horizontal dash line represents the *p*-value threshold for FDR < 0.05. The red dots denote miRNAs that meet both FC ≥ 1.2 and FDR < 0.05 criteria, while light blue dots meet FDR < 0.05 but not FC ≥ 1.2, and green dots meet FC ≥ 1.2 but not FDR < 0.05 criteria.

Among the 2,652 miRNA genes quantified, 834 genes had count per million reads (cpm) greater than 0.25 in at least 50% of the samples and were carried forward for differential gene expression analysis. Four and 64 miRNAs were differentially expressed between AD cases and cognitively normal controls in the IFG and STG, respectively (volcano plots in Figure 1). The full list of DEGs with adjusted *p*-value less than 0.05 is listed in Table 2.

**TABLE 2:** A list of differentially expressed miRNA associated with AD from this study (FDR adjusted *p*-value less than 0.05).

**miRNA**	**IFG**				**STG**			
	**logFC**	**AveExpr**	***P*.value**	**adj.*P*.val**	**logFC**	**AveExpr**	***P*.value**	**adj.*P*.val**
hsa-miR-132-5p	−0.74	7.40	1.61E-07	6.72E-05	−0.66	7.40	7.96E-07	4.81E-04
hsa-miR-212-5p	−0.90	5.65	8.39E-08	6.72E-05	−0.60	5.65	1.13E-04	4.71E-03
hsa-miR-132-3p	−0.79	7.72	1.80E-06	5.01E-04	−0.23	7.72	1.21E-01	3.62E-01
hsa-miR-212-3p	−0.69	5.25	5.11E-05	1.06E-02	−0.31	5.25	4.49E-02	2.05E-01
hsa-miR-502-3p	0.02	5.30	8.14E-01	9.96E-01	0.40	5.30	1.15E-06	4.81E-04
hsa-miR-500a-3p	0.02	5.91	7.92E-01	9.96E-01	0.35	5.91	3.66E-06	1.02E-03
hsa-miR-320a-3p	0.11	10.05	1.71E-01	7.91E-01	0.34	10.05	8.89E-06	1.06E-03
hsa-miR-320b	0.10	10.40	2.19E-01	8.24E-01	0.36	10.40	6.35E-06	1.06E-03
hsa-miR-95-3p	−0.10	8.56	2.57E-01	8.24E-01	0.37	8.56	7.88E-06	1.06E-03
hsa-miR-1-3p	0.13	8.94	4.62E-01	9.19E-01	0.76	8.94	6.56E-06	1.06E-03
hsa-miR-148b-3p	−0.05	9.68	3.72E-01	8.53E-01	0.24	9.68	2.60E-05	2.41E-03
hsa-miR-4510	0.07	0.98	5.02E-01	9.27E-01	0.40	0.98	2.53E-05	2.41E-03
hsa-miR-6505-3p	−0.08	−0.32	5.95E-01	9.72E-01	−0.61	−0.32	3.00E-05	2.51E-03
hsa-miR-421	−0.18	6.34	9.51E-03	3.93E-01	−0.28	6.34	5.07E-05	3.02E-03
hsa-miR-199a-3p	0.07	7.58	4.55E-01	9.17E-01	0.39	7.58	4.24E-05	3.02E-03
hsa-miR-199b-3p	0.07	6.58	4.54E-01	9.17E-01	0.39	6.58	4.41E-05	3.02E-03
hsa-miR-628-3p	−0.03	4.55	7.61E-01	9.96E-01	−0.36	4.55	5.00E-05	3.02E-03
hsa-miR-4446-3p	−0.23	2.11	8.35E-02	6.46E-01	−0.50	2.11	7.95E-05	4.28E-03
hsa-miR-146a-5p	0.04	8.40	6.33E-01	9.82E-01	0.29	8.40	8.21E-05	4.28E-03
hsa-miR-4742-3p	−0.29	0.01	1.21E-02	4.14E-01	−0.43	0.01	8.81E-05	4.32E-03
hsa-let-7g-5p	−0.07	13.94	1.97E-01	7.99E-01	0.19	13.94	1.12E-04	4.71E-03
hsa-miR-501-3p	0.02	5.74	8.29E-01	9.96E-01	0.27	5.74	1.10E-04	4.71E-03
hsa-miR-500b-5p	0.03	0.51	7.77E-01	9.96E-01	0.41	0.51	1.35E-04	5.37E-03
hsa-miR-32-5p	−0.03	5.12	7.23E-01	9.96E-01	−0.28	5.12	1.62E-04	6.13E-03
hsa-miR-22-5p	−0.03	5.98	6.48E-01	9.88E-01	−0.26	5.98	2.03E-04	7.23E-03
hsa-miR-500a-5p	0.03	0.50	7.56E-01	9.96E-01	0.40	0.50	2.08E-04	7.23E-03
hsa-miR-629-5p	0.09	4.61	1.69E-01	7.91E-01	0.22	4.61	2.43E-04	8.10E-03
hsa-miR-30c-2-3p	0.04	5.42	5.99E-01	9.72E-01	0.27	5.42	2.53E-04	8.11E-03
hsa-miR-10395-3p	0.03	3.15	8.79E-01	9.96E-01	0.75	3.15	2.80E-04	8.65E-03
hsa-miR-664a-3p	0.02	3.31	8.22E-01	9.96E-01	0.34	3.31	5.10E-04	1.52E-02
hsa-miR-34a-5p	0.10	5.25	5.64E-01	9.60E-01	0.60	5.25	5.41E-04	1.56E-02
hsa-miR-6882-5p	0.06	0.21	5.60E-01	9.60E-01	0.35	0.21	5.81E-04	1.61E-02
hsa-miR-30e-3p	0.06	9.70	2.97E-01	8.24E-01	0.18	9.70	6.44E-04	1.73E-02
hsa-miR-378a-3p	0.09	8.00	1.66E-01	7.91E-01	0.22	8.00	6.73E-04	1.75E-02
hsa-miR-339-5p	−0.19	3.41	4.18E-02	5.61E-01	−0.30	3.41	7.85E-04	1.89E-02
hsa-miR-378d	0.17	−1.35	3.19E-01	8.26E-01	0.57	−1.35	7.57E-04	1.89E-02
hsa-miR-548e-3p	0.06	1.16	4.72E-01	9.19E-01	0.27	1.16	7.94E-04	1.89E-02
hsa-miR-126-5p	−0.07	7.36	4.89E-01	9.26E-01	0.33	7.36	9.29E-04	2.15E-02
hsa-miR-514a-3p	−0.17	3.88	3.25E-01	8.26E-01	−0.55	3.88	9.80E-04	2.21E-02
hsa-miR-222-5p	0.24	−0.80	1.94E-01	7.99E-01	0.57	−0.80	1.07E-03	2.31E-02
hsa-miR-99b-5p	−0.01	12.04	8.11E-01	9.96E-01	0.17	12.04	1.08E-03	2.31E-02
hsa-miR-1277-3p	−0.11	0.72	4.48E-01	9.13E-01	−0.45	0.72	1.16E-03	2.41E-02
hsa-miR-28-3p	0.02	6.22	8.06E-01	9.96E-01	0.23	6.22	1.25E-03	2.54E-02
hsa-miR-128-1-5p	−0.13	3.85	3.24E-01	8.26E-01	0.40	3.85	1.31E-03	2.59E-02
hsa-miR-487a-5p	−0.12	5.15	3.61E-01	8.52E-01	−0.39	5.15	1.64E-03	3.17E-02
hsa-miR-3912-5p	0.09	1.85	2.80E-01	8.24E-01	0.26	1.85	1.70E-03	3.22E-02
hsa-miR-30e-5p	0.00	12.48	9.67E-01	9.99E-01	0.17	12.48	1.80E-03	3.34E-02
hsa-miR-30a-3p	0.05	9.54	3.43E-01	8.38E-01	0.17	9.54	1.87E-03	3.38E-02
hsa-miR-362-3p	−0.09	−1.60	6.02E-01	9.72E-01	0.53	−1.60	2.09E-03	3.70E-02
hsa-miR-185-5p	−0.05	9.00	3.71E-01	8.53E-01	0.18	9.00	2.19E-03	3.81E-02
hsa-miR-454-3p	−0.09	3.72	2.43E-01	8.24E-01	0.23	3.72	2.43E-03	4.09E-02
hsa-miR-301a-5p	−0.05	3.49	3.27E-01	8.26E-01	−0.15	3.49	2.45E-03	4.09E-02
hsa-miR-433-3p	−0.16	9.33	5.50E-02	6.13E-01	−0.24	9.33	2.59E-03	4.24E-02
hsa-miR-3912-3p	0.10	2.16	2.23E-01	8.24E-01	0.24	2.16	2.80E-03	4.48E-02
hsa-miR-3151-5p	0.03	0.97	7.62E-01	9.96E-01	−0.32	0.97	2.90E-03	4.48E-02
hsa-miR-381-5p	0.03	−0.99	8.56E-01	9.96E-01	0.53	−0.99	2.86E-03	4.48E-02
hsa-miR-323a-5p	−0.45	3.43	1.59E-02	4.28E-01	−0.53	3.43	3.38E-03	4.61E-02
hsa-miR-708-3p	0.10	5.31	1.29E-01	7.69E-01	0.19	5.31	3.26E-03	4.61E-02
hsa-miR-133b	0.20	3.06	2.53E-01	8.24E-01	0.50	3.06	3.34E-03	4.61E-02
hsa-miR-582-3p	−0.07	6.07	4.05E-01	8.86E-01	−0.24	6.07	3.28E-03	4.61E-02
hsa-miR-1180-3p	0.05	7.88	5.96E-01	9.72E-01	−0.26	7.88	3.33E-03	4.61E-02
hsa-miR-301b-3p	0.05	0.01	6.61E-01	9.88E-01	−0.34	0.01	3.35E-03	4.61E-02
hsa-miR-543	0.02	7.75	8.51E-01	9.96E-01	−0.28	7.75	3.10E-03	4.61E-02
hsa-miR-6505-5p	−0.17	1.53	8.86E-02	6.46E-01	−0.29	1.53	3.54E-03	4.76E-02
hsa-miR-191-3p	−0.12	2.47	1.70E-01	7.91E-01	−0.24	2.47	3.79E-03	4.96E-02
hsa-miR-9-5p	0.00	17.51	9.19E-01	9.96E-01	0.13	17.51	3.80E-03	4.96E-02

**FIGURE 2:**
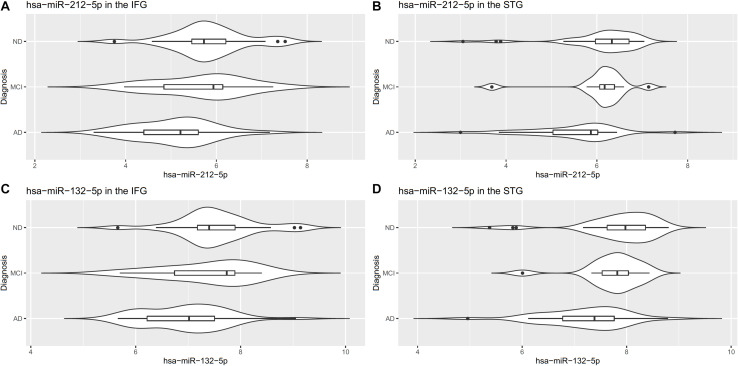
Differentially expressed miR-212-5p in the IFG **(A)** and STG **(B)** and miR-132-5p in the IFG **(C)** and STG **(D)**.

Notably, two miRNAs (hsa-miR-212-5p and hsa-miR-132-5p) were significantly differentially expressed in both brain regions, and their counterparts,hsa-miR-212-3p and hsa-miR-132-3p,were significantly differentially expressed in the IFG. In the IFG, hsa-miR-212-5p/-3p were significantly down-regulated in AD samples compared to cognitively normal controls (hsa-miR-212-5p, FDR adjusted *p*-value = 6.72 × 10^–5^, Figure 2A; hsa-miR-212-3p, adjusted *p*-value = 0.01). The same trend was observed in the STG for hsa-miR-212-5p (adjusted *p*-value = 4.71 × 10^–4^, Figure 2B). hsa-miR-132-5p/-3p were down-regulated in AD compared to cognitively normal controls in the IFG (hsa-miR-132-5p, FDR adjusted *p*-value = 6.72 × 10^–5^, Figure 2C; hsa-miR-132-3p, adjusted *p*-value = 1.80 × 10^–6^), and hsa-miR-132-5p was also down-regulated in the STG (adjusted *p*-value = 4.81 × 10^–4^, Figure 2D). In addition, hsa-miR-146a-5p, hsa-miR-501-3p, hsa-miR-34a-5p, and hsa-miR-454-3p were significantly up-regulated in AD compared to cognitively normal controls (adjusted *p* < 0.05, see Table 2 and Supplementary Table 2A for test statistics). These miRNAs and hsa-miR-132-5p were reported to be either strongly or suggestively associated with AD in the brain in the recent meta-analysis (Takousis et al., 2019) with consistent directionality. The results from this study for the 25 brain miRNA described in the recent meta-analysis (Takousis et al., 2019) are shown in Supplementary Table 2A, including 8 other nominally differentially expressed miRNA with nominal *p*-value less than 0.05 in at least one of the brain regions in this study. Significantly differentially expressed miRNAs in AD that have been previously reported and hypothesized to play roles in Ab, tau, neuroinflammation, and cell death/phagocytosis are highlighted in Table 3. miRNAs with nominal *p*-values less than 0.05 involved in these biological processes or reported previously in peripheral blood, CSF, or brain are also highlighted in Supplementary Table 2B.

**TABLE 3:** Significant differentially expressed miRNA previously implicated in AD compared to cognitively normal controls.

**miRNA**	**IFG**			**STG**			**Biological relevance**	**References**
		**FC**	***p*-value**		**FC**	***p*-value**		
**Amyloid hypothesis**								
hsa-miR-339-5p	–	−0.19	4.18E-02	–	−0.30	7.85E-04	Was reported to down-regulate *BACE1* expression and was reported to be down-regulated in AD brain	Long et al., 2014
hsa-let-7g-5p		−0.07	1.97E-01	++	0.19	1.12E-04	*C. elegans* homolog of APP, APP-like-1 (apl-1) showed significant genetic interactions with let-7 family of microRNAs and let-7-targeted heterochronic genes. The level of the apl-1 transcription was modulated by the activity of let-7 family of microRNAs. Reported to be down-regulated in blood in 2 studies	Niwa et al., 2008; Nagaraj et al., 2019
**Tau hypothesis**								
hsa-miR-34a-5p		0.10	5.64E-01	++	0.60	5.41E-04	miR-34a was showed to repress the expression of endogenous tau protein in human neuroblastoma cell line M17D cells. Conversely, inhibition of endogenously expressed miR-34 family members leaded to increased endogenous tau expression. hsa-miR-34a-5p was identified as suggestively up-regulated (*p* = 1.89 × 10^–7^) in Takousis et al. (2019) meta-analysis.	Dickson et al., 2013; Takousis et al., 2019
hsa-miR-212-5p	–	−0.90	8.39E-08	–	−0.60	1.13E-04	miR-132/212 deficiency in mice leads to increased tau expression, phosphorylation and aggregation.	Smith et al., 2015
hsa-miR-212-3p	–	−0.69	5.11E-05	–	−0.31	4.49E-02		
hsa-miR-132-5p	–	−0.74	1.61E-07	–	−0.66	7.96E-07	miR-132/212 deficiency in mice leads to increased tau expression, phosphorylation and aggregation. miR-132 directly targets tau mRNA to regulate its expression. miR-132 plays a role in neurogenesis and neuronal differentiation. hsa-miR-132-5p was identified as suggestively down-regulated (*p* = 3.01 × 10^–10^) in Takousis et al. (2019) meta-analysis	Lukiw, 2007; Smith et al., 2015
hsa-miR-132-3p	–	−0.79	1.80E-06		−0.23	1.21E-01		
**Immune-related**								
hsa-miR-146a-5p		0.04	6.33E-01	++	0.29	8.21E-05	miR-146a targets IL-1 receptor-associated kinase 1 (IRAK1) and TNF receptor-associated factor 6 (TRAF6) which are key adapter molecules in TLR and IL-1 receptor signaling cascades, mediates activation of NF-kB and AP-1 pathways. miR-146a also down-regulates complement factor H (CFH), an important repressor of the inflammatory response in the brain, highlighting the inflammatory component in the AD pathogenesis. [11] hsa-miR-146a-5p was identified as suggestively up-regulated (*p* = 4.88 × 10^–7^) in Takousis et al. (2019) meta-analysis. Herrera-Espejo et al. (2019) reported that hsa-miR-146a-5p was mostly up-regulated. Reported to be down-regulated in blood in 2 studies by Nagaraj et al. (2019)	Taganov et al., 2006; Lukiw et al., 2008; Nagaraj et al., 2019; Takousis et al., 2019
**Cell death/phagocytosis**								
hsa-miR-128-1-5p		−0.13	3.24E-01	++	0.40	1.31E-03	Down-regulates lysosomal enzymes and upregulated in monocytes of AD patients; hsa-miR-128-3p was identified as suggestively up-regulated in blood (*p* = 1.53 × 10^–6^) in Takousis et al. (2019) meta-analysis	Lukiw, 2007; Takousis et al., 2019
**Other prior corroborating evidence**								
hsa-miR-454-3p		−0.09	2.43E-01	++	0.23	2.43E-03	hsa-miR-454-3p was identified as strongly up-regulated in brain (*p* = 4.86 × 10^–6^) in Takousis et al. (2019) meta-analysis	Takousis et al., 2019
hsa-miR-502-3p		0.02	8.14E-01	++	0.40	1.15E-06	Reported previously to be up-regulated in plasma and down-regulated in blood. Discordant directionality of dysregulation as reviewed by Nagaraj et al. (2019)	Satoh et al., 2015; Nagaraj et al., 2017
hsa-miR-9-5p		0.00	9.19E-01	++	0.13	3.80E-03	Discordant directionality of dysregulation in blood and down-regulated in CSF as reviewed by Nagaraj et al. (2019)	Nagaraj et al., 2019
hsa-miR-501-3p		0.02	8.29E-01	++	0.27	1.10E-04	Serum hsa-miR-501-3p levels were downregulated in AD patients, and its lower levels significantly correlated with lower Mini-Mental State Examination scores. Contrary to its serum levels, hsa-miR-501-3p was remarkably upregulated in the same donors’ AD brains. hsa-miR-501-3p was identified as strongly up-regulated (*p* = 2.03 × 10^–11^) in Takousis et al. (2019) meta-analysis.	Hara et al., 2017; Takousis et al., 2019
hsa-miR-30a-3p		0.05	3.43E-01	++	0.17	1.87E-03	Reported previously to be down-regulated in CSF by Lusardi et al. (2017) but up-regulated in CSF by Cogswell et al. (2008). Discordant directionality of dysregulation as reviewed by Nagaraj et al. (2019)	Cogswell et al., 2008; Lusardi et al., 2017

*+ ⁣ + denotes differentially up-regulated miRNA with FDR adjusted p-value less than 0.05, while +/− denotes nominally up-/down-regulated miRNA with p-value less than 0.05.*

The targets of the differentially expressed miRNAs were then evaluated. Differentially expressed miRNA (adjusted *p*-value < 0.1) with high-confidence experimental or predicted targets (CWCS as defined by TargetScan v7.2 is −0.4 or lower) from Ingenuity Knowledge Base and nominally differentially expressed in the opposite direction in the paired mRNA-Seq samples (*p* < 0.05) were prioritized (Supplementary Tables 3A,B).

There were 1,267 such miRNA-mRNA pairs from the STG analysis, 164 of which were experimentally observed, and most of the evidence was from TargetScan v7.2. The comprehensive lists of miRNA-mRNA pairs with miRNA and mRNA differentially gene expression evidence are available in Supplementary Tables 3A,B for the STG and IFG analysis, respectively. Additional candidate targets from TargetScan v7.2 and v6.2 with miRNA-mRNA correlation passing multiple testing thresholds are available in Supplementary Tables 4A,B for the STG analysis.

Seven miRNAs were associated with Braak stage in the IFG, while no significant miRNA was associated with Braak stage in the STG (Table 4 and Supplementary Figure 1). When pooling both STG and IFG samples together, 12 miRNAs including hsa-miR-212-5p (adjusted *p*-value = 1.79 × 10^–4^), hsa-miR-132-5p (adjusted *p*-value = 3.42 × 10^–4^), and hsa-miR-132-3p (adjusted *p*-value = 2.59 × 10^–3^) were identified to be associated with the Braak neurofibrillary stage with FDR adjusted *p*-value less than 0.05 in a model that also corrected for brain region. Although the signal was weaker when analyzing separately for each brain region, the associations between hsa-miR-212-5p (adjusted *p*-value = 3.14 × 10^–2^), hsa-miR-132-3p (adjusted *p*-value = 4.67 × 10^–2^) and Braak stage in the IFG was still significant, while hsa-miR-132-5p (*p* = 1.39 × 10^–4^, adjusted *p*-value = 5.70 × 10^–2^) was nominally associated with Braak stage in the STG. A full list of miRNAs with FDR adjusted *p*-value less than 0.1 is shown in Supplementary Table 5.

**TABLE 4:** A list of differentially expressed miRNA associated with pathology from this study (FDR adjusted p-value less than 0.05).

**miRNA**	**logFC**	**AveExpr**	***t***	***P*.value**	**adj.*P*.val**
**IFG**					
hsa-miR-152-5p	−0.18	−1.07	−4.29	5.83E-05	3.14E-02
hsa-miR-296-3p	−0.12	1.95	−4.13	1.02E-04	3.14E-02
hsa-miR-212-5p	−0.22	5.41	−4.10	1.13E-04	3.14E-02
hsa-miR-210-3p	−0.15	3.83	−3.93	2.00E-04	3.57E-02
hsa-miR-4758-5p	0.23	−2.11	3.90	2.23E-04	3.57E-02
hsa-miR-1250-5p	−0.13	4.32	−3.86	2.57E-04	3.57E-02
hsa-miR-132-3p	−0.19	7.24	−3.73	3.92E-04	4.67E-02
**Samples from all brain regions combined, but the statistical model additionally corrected for brain region**					
hsa-miR-212-5p	−0.19	5.65	−5.43	2.14E-07	1.79E-04
hsa-miR-5701	0.40	1.23	5.07	1.16E-06	3.42E-04
hsa-miR-132-5p	−0.16	7.40	−5.05	1.23E-06	3.42E-04
hsa-miR-132-3p	−0.13	7.72	−4.52	1.24E-05	2.59E-03
hsa-let-7e-3p	0.07	4.42	4.46	1.56E-05	2.60E-03
hsa-miR-187-3p	0.07	2.76	4.16	5.19E-05	7.17E-03
hsa-miR-383-5p	0.07	7.17	4.13	6.02E-05	7.17E-03
hsa-miR-302a-5p	0.15	0.66	4.01	9.59E-05	9.99E-03
hsa-miR-323b-3p	−0.12	7.65	−3.92	1.34E-04	1.24E-02
hsa-miR-222-5p	0.14	−0.80	3.80	2.08E-04	1.74E-02
hsa-miR-10395-3p	0.16	3.15	3.59	4.40E-04	3.34E-02
hsa-miR-30e-3p	0.04	9.70	3.50	6.04E-04	4.20E-02

Over-representation analysis revealed that these targets are enriched in the Wnt signaling pathway (FDR *q*-value = 1.06 × 10^–12^), MAPK family signaling cascades (FDR *q*-value = 9.39 × 10^–8^), sphingosine 1-phosphate (S1P) pathway (FDR *q*-value = 1.28 × 10^–6^), adaptive immune system (FDR *q*-value = 5.98 × 10^–12^), innate immune system (FDR *q*-value = 2.97 × 10^–10^), neurogenesis (FDR *q*-value = 1.25 × 10^–30^). A full list of the overrepresented gene sets is available in Supplementary Table 6.

## Discussion

Previous studies across brain regions suggest that transcriptional changes across different regions of the brain occur at different stages of AD. A few regions, including the STG, located in the temporal lobe and responsible for auditory processing, have high levels of differentially expressed transcripts during mild cognitive impairment. Other regions, including the IFG, located in the frontal lobe and responsible for language processing and production, differentially expressed transcripts are low during the early stages of Alzheimer’s but gradually increase as the disease progresses (Haroutunian et al., 2009). Our results are consistent with these observations as we observe 64 differentially expressed miRNAs between cognitively normal and AD samples in the STG, while only four miRNAs were significantly different in the IFG.

Two miRNAs (miR-212-5p and miR-132-5p) were consistently down-regulated between both tissues, while miR-212-3p and miR-132-3p were down-regulated in the IFG. miR-212-5p, miR-132-5p, and miR-132-3p were additionally associated with AD pathology as captured by Braak stage. We replicated observations from four additional miRNAs (hsa-miR-146a-5p, hsa-miR-501-3p, hsa-miR-34a-5p, and hsa-miR-454-3p, adjusted *p* < 0.05) previously reported in a meta-analysis to be strongly or suggestively associated with AD in the brain and provide supporting evidence for an additional 8 miRNAs (*p* < 0.05) although they did not reach study-wide significance in this study. We have also identified novel miRNAs to be associated with AD from this study, including hsa-miR-4446-3p. Future replication of these miRNAs as well as experimentally verification of their targets are warranted.

Among the miRNAs identified, several biological pathways and processes have been implicated including cell death/autophagy, immune related pathways, Ab and tau pathogenesis.

### Amyloid Hypothesis

The amyloid b-peptide (Ab) is a hallmark of AD and is produced by sequential proteolytic cleavages of the amyloid precursor protein (APP) by b- and -g secretases (Tanzi and Bertram, 2005). APP is also processed in a non-amyloidogenic pathway by a-secretase, thereby repressing Ab formation (Postina et al., 2004). Several miRNAs involved in Ab processing were identified in this study, including miR-339-5p, which was significantly down-regulated in the STG (FDR adjusted-*p*-value = 0.02) and nominally down-regulated in the IFG, consistent with the down-regulation reported in the literature (Long et al., 2014). The miR-339-5p target site is predicted to be in the 3′-UTR of *BACE1*, which encodes b-secretases. Co-transfection of miR-339-5p with a *BACE1* 3′-UTR reporter construct resulted in significant reduction in reporter expression, confirming the *in silico* prediction that the *BACE1* transcript is the target of miR-339-5p (Long et al., 2014). Hsa-miR-221-3p and hsa-miR-144-3p (Supplementary Tables 1, 2B) were nominally down-regulated in the IFG and STG, respectively, consistent with a report that miR-144-5p and miR-221 are down-regulated in AD (Manzine et al., 2018). Over-expression of miR-144 and miR-221 has been reported to significantly decrease activity of *ADAM10*, which encodes a-secretase, in the *ADAM10* 3′-UTR reporter assay (Cheng et al., 2013; Manzine et al., 2018). Additional genetic interactions between the miRNA hsa-let-7g-5p and *APP* in the model system *Caenorhabditis elegans* is discussed in the Supplementary Text. Additional miRNAs associated with AD include miR-132 and miR-212, which are downregulated across the IFG and STG in AD samples compared to cognitively normal samples. Loss of miR-132 and miR-212 in triple transgenic AD background mice was previously demonstrated to promote Ab pathology and amyloid plaque formation (Hernandez-Rapp et al., 2016).

### Tau Hypothesis

miR-132 and miR-212 are located as a gene cluster in cytoband 17p13.3. miR-132 and tau co-express in the same neuronal populations (Lau et al., 2013), and miR-132 directly targets tau mRNA at the 3′ UTR to regulate its expression (Smith et al., 2015). miR-132 may also regulate tau alternative splicing *in vitro* by targeting poly-pyrimidine tract-binding protein 2 (*PTBP2*), and its levels correlate with tau splicing defects in patients with progressive supranuclear palsy (PSP) cases (Smith et al., 2011). miR-132/212 deficiency in mice leads to increased tau expression, phosphorylation and aggregation (Smith et al., 2015). Furthermore, downregulation of miR-132 and its paralog miR-212 disturbs the balance of *S*-nitrosylation and induces tau phosphorylation in a neuronal nitric oxide synthase (NOS1)-dependent manner and miR-132/212 directly regulated the expression of NOS1 (Wang et al., 2017). Deletion of miR-132/212 induces tau aggregation in mice expressing endogenous or human mutant tau, an effect associated with autophagy dysfunction. Conversely, treatment of AD mice with miR-132 mimics has been found to restore memory function and tau metabolism (Smith et al., 2015). miR-132 and miR-212 levels were also reported to be correlated with insoluble tau and cognitive impairment in humans (Smith et al., 2015) and with the severity of tau pathology (Wang et al., 2011; Lau et al., 2013).

### Novel Findings

We also have identified novel miRNAs associated with AD (Table 2), including hsa-miR-4446-3p which was identified in the STG (adjusted *p*-value = 0.004). Based on Ingenuity Knowledge Base (including both conserved and non-conserved sites from TargetScan v7.2), hsa-miR-4446-3p has several predicted high confidence targets that were differentially expressed in the opposite direction of the miRNA. These include cytochrome c, somatic (*CYCS*); interferon, lambda receptor 1 (*IFNLR1*)*;* and synaptotagmin II (*SYT2*) from TargetScan (Supplementary Table 3C). *CYCS* is involved in apoptosis signaling and mitochondrial dysfunction; *IFNLR1* is involved in PI3K/AKT signaling and STAT3 pathway; and *SYT2* is involved in synaptogenesis signaling pathway. These pathways have previously been implicated in AD. When the list expands to include potential targets with conserved sites from the TargetScan database v6.2 with context scores less than −0.2 (equivalent to Ingenuity Knowledge Base’s moderate confidence targets, Supplementary Table 4B), additional genes such as *ZNF385A*, *USF2*, and *AUP1* were not only significantly differentially expressed in the paired STG mRNA samples (FDR adjusted *p*-value < 0.05), but also had sample-wise negative correlation between miRNA level and mRNA transcript level (Supplementary Figure 2 and Supplementary Table 4B). Surprisingly, hsa-miR-4446-3p was positively correlated with *MAPT* (Spearman’s rank correlation *r* = 0.45, *p*-value = 4.91 × 10^–9^). The effect size of this correlation is moderate according to Cohen’s convention on interpretation of correlation metrics (Cohen, 1988). The biological implication of this observation is unknown as a negative correlation is expected for miRNAs is thought to regulate gene expression at the post-transcriptional level by base-pairing with 3′-untranslated region (3′-UTR) of the target gene, causing cleavage/degradation of the cognate mRNA or preventing translation initiation. However, a positive correlation could be expected if there are 2 levels of consecutive negative correlation.

miR-4446-3p has been reported to be up-regulated by (tumor growth-generated) mechanical compression in both MDA-MB-231 and cancer-associated fibroblasts (CAFs), while more than 25% of down regulated target genes were functionally involved in tumor suppression (apoptosis, cell adhesion, and cell cycle arrest) in a parallel mRNA array analysis (Kim et al., 2016). In the brain of AD patients, there is also an analogous microenvironment with neurons interacting with amyloid plaques/neurofibril tangles similar to tumor and stromal cell interaction. More experiments are needed to shed light on the role of miR-4446-3p.

miRNAs seems to be promising biomarkers for early detection of disease, risk assessment, and prognosis especially if detected in non-invasive tissue sources such as peripheral blood cells, exosomes or body fluid such as serum, plasma, or cerebrospinal fluid (CSF) (Kumar and Reddy, 2016; Espinosa-Parrilla et al., 2019; Herrera-Espejo et al., 2019; Zhang et al., 2019). Zhang et al. (2019) evaluated the diagnostic characteristics of peripheral blood miRNAs for AD diagnosis from 10 studies containing 770 AD and 664 normal controls and concluded that overall sensitivity of 0.80, specificity of 0.83, and diagnostic odds ratio of 14 (Zhang et al., 2019). Even though this study does not address AD biomarkers directly, we nominate under-studied miRNAs for further examination in peripheral tissues for their possible diagnostic utility in lower cost non-invasive AD diagnosis and AD progression prediction.

There are several limitations of the study. The average age of the AD samples was older than those of cognitively normal controls. To preserve the power of the study, we chose to correct for age in the statistical model rather than only analyze aged-match samples. The sample sizes for the MCI samples are smaller than the other two diagnosis groups and therefore they were only used in the graphical displays and those results shall be interpreted with cautions.

In our study, we replicated known miRNAs involved in AD but also identified novel miRNAs implicated in AD etiology and nominated targets for miRNAs for future experimental confirmation and replication.

## Data Availability Statement

The data presented in the study are deposited in the NCBI repository, accession number PRJNA670793.

## Ethics Statement

The studies involving human participants were reviewed and approved by WIRB-Copernicus Group, Inc. The patients/participants provided their written informed consent to participate in this study.

## Author Contributions

QSL conceived the project, designed the study, and generated the study data. QSL and DC undertook the data analysis and bioinformatics. QSL drafted the first draft of the manuscript. All authors provided feedback and approved the final submission.

## Conflict of Interest

QL and DC are employees of Janssen Research & Development, LLC and may own stock/stock options in the company.
