# Supplementary material for: Integrated miRNA-Seq and mRNA-Seq Study to Identify miRNAs Associated With Alzheimer’s Disease Using Post-mortem Brain Tissue Samples
Source: Front Neurosci. 2021 Mar 23;15:620899. doi: 10.3389/fnins.2021.620899 (PMC8021900; doi:10.3389/fnins.2021.620899)
Supplement: Supplementary file 2 [file Data_Sheet_1.docx]

Integrated miRNA-Seq and mRNA-Seq study to identify miRNAs associated Alzheimer’s disease using post-mortem brain tissue samples

Qingqin S Li^1,*^, and Diana Cai^1,a^

^1^Neuroscience, Janssen Research & Development, LLC, Titusville, NJ

^a^Current affiliation: Discovery Science, Janssen Research & Development, LLC, Boston, MA

*** Correspondence:**

Qingqin S Li, Ph.D.

[qli2@its.jnj.com](mailto:qli2@its.jnj.com)

**Supplemental Text 1** Explanation of Ingenuity Knowledge Base

**Supplemental Text 2** Discussion of miRNAs identified in this study in the context of literature

**Supplemental Table 1** A list of nominally significant differentially expressed miRNA from this study (p-value less than 0.05)

**Supplemental Table 2A** Significantly or nominally differentiated expressed miRNA previously implicated in AD experimentally, or reported to be differentially expressed in previously (not an exhaustive search) or identified in the Takousis et al., meta-analysis study.

**Supplemental Table 2B** Brain miRNAs identified in Takousi et al., 2019 Meta-Analysis and their results from this study

**Supplemental Table 3A & 3B** Differentially expressed miRNA comparing AD vs. cognitively normal controls (FDR adjusted p-value less than 0.1) from the STG (A) and IFG (B) with its targets (experimentally observed or predicted with high confidence) differentially expressed in opposite direction in the paired mRNA-Seq data

**Supplemental Table 3C & 3D** Differentially expressed miRNA comparing AD vs. cognitively normal controls (FDR adjusted p-value less than 0.1) from the (C) STG (D) IFG with its targets (experimentally observed or predicted with high confidence) in opposite direction in the paired mRNA-Seq data

**Supplemental Table 3E** Experimentally observed or predicted with high confidence targets for differentially expressed miRNA associated with Braak stage (FDR adjusted p-value less than 0.1)

**Supplemental Table 3F & 3G** Experimentally observed or predicted with high confidence targets for differentially expressed miRNA associated with Braak stage in the IFG (F) and STG (G) (FDR adjusted p-value less than 0.1)

**Supplemental Table 4A** A list of putative targets (from TargetScan 7.2 conserved and non-conserved sites with weighted context score < -0.2) of differentially expressed miRNAs in the STG with (anti-)correlated mRNA passing multiple testing correction threshold (correcting for all possible miRNA-mRNA pairs, e.g. p < 0.05/2672~1.871257e-05)

**Supplemental Table 4B** A list of putative targets (from TargetScan 6.2 Conserved sites with context score < -0.2) of differentially expressed miRNAs in the STG with (anti-)correlated mRNA passing multiple testing correction threshold (correcting for all possible miRNA-mRNA pairs, e.g. p < 0.05/25496~1.961092e-06)

**Supplemental Table 5** A list of miRNA associated with Braak neurofibrillary stage with FDR adjust p-value less than 0.1

**Supplemental Table 6** Over-representation analysis of the gene list from Supplemental Table S3A and S3B

**Supplemental Figure 1** Association with Braak stage for hsa-miR212-5p in the IFG (A), STG (B), hsa-miR-132-5p in the in the IFG (C) and STG (D)

**Supplemental Figure 2** Negative correlation between hsa-miR-4446-3p and (A) *ZNF385A* (B) *UBE2Q1* (C) *USF2* and (D) *AUP1*

**Supplemental Figure 3** Anti-correlation between hsa-miR-592 and hsa-miR-148b-3p

**Supplemental Text 1**: Explanation of Ingenuity Knowledge Base

Supplemental Table 3 included predicted miRNA targets and experimentally observed miRNA targets captured by Ingenuity Knowledge Base. Please see below for some related documents from Ingenuity.

**Source:** **Confidence Level Filter - microRNA** https://qiagen.secure.force.com/KnowledgeBase/KnowledgeIPAPage?id=kA41i000000L5ptCAC

**“Confidence Level Filter for miRNA targeting**

This filter allows you to select miRNA-mRNA targeting relationships based on whether you want to choose experimentally verified or predicted. The databases used for both predicted and experimentally verified targets are indicated below.  The confidence level of high and moderate for predicted targets from target scan is as defined by TargetScan

**TargetScan Predicted microRNA-mRNA Target Interactions**

Over 1.3 million predicted microRNA-mRNA target relationships from TargetScan were added to the Ingenuity Knowledge Base.
The confidence levels of predictions estimated based on TargetScan’s published methodology and organized into High and Moderate confidence for use in the microRNA Target Filter

- High confidence assigned when the cumulative weighted context score (or “CWCS”) as defined by TargetScan is -0.4 or lower. Such scores predict that a microRNA represses a particular mRNA target by at least 25% relative to the normal level.
- Moderate confidence is assigned when CWCS is between -0.2 and -0.4. Such scores predict that a microRNA represses a particular mRNA target between 13% and 25% compared to their normal levels.

To compute the amount of expected repression yourself:

- Repression ratio = - (2CWCS -1).
  - For example a CWCS of -0.467 equates to expected repression of 0.28, or 28%
  - Repression ratio formula in Excel:  = - (POWER(2,-0.467) -1)

**Tarbase, miRecords and Ingenuity Curated** **microRNA-mRNA Target Interactions** 

TarBase is a database which houses a manually curated collection of experimentally supported microRNA (miRNA) targets in several animal species of central scientific interest, plants and viruses. The current version includes more than 1300 experimentally supported targets. Currently, only the mammalian targeting interactions from TarBase are included in IPA.

miRecords represents the largest database of experimentally validated microRNA targeting interactions from human, mouse and rat (as well as other species not included in IPA). The current version of miRecords contains over 2,600 experimentally validated interactions from human, mouse and rat.”

**Source: microRNA Target Filter FAQ** https://qiagen.secure.force.com/KnowledgeBase/KnowledgeIPAPage?id=kA41i000000L5phCAC

**“8. Does IPA use TarBase (validated) targets?**

IPA has several sources of experimentally determined microRNA targeting information, namely miRecords, TarBase, and direct acquisition from the literature by our curation scientists (Ingenuity Expert Findings).

TargetScan provides our predicted microRNA-mRNA targeting content. TargetScan is a software provided by MIT for the prediction of miRNA targets to mRNA. In TargetScan, miRNA are paired with “Target Genes” which are mRNAs. Approximately 1.3 million predicted miRNA-mRNA binding relationships have been imported from TargetScan into IPA.”

**Source:** **Clustering of miRNA in IPA https://qiagen.secure.force.com/KnowledgeBase/KnowledgeIPAPage?id=kA41i000000L5poCAC**

**Supplemental Text 2** Discussion of miRNAs identified in this study in the context of literature

Additional miRNAs of interest are further discussed below.

Amyloid hypothesis

In model system *C. elegans*, the timing of cell fate determination is controlled by the heterochronic genes, including let-7 family of microRNAs. C. elegans homolog of *APP*, APP-like-1 (*apl-1*) shows significant genetic interactions with let-7 family of microRNAs and let-7-targeted heterochronic genes, *hbl-1, lin-41 and lin-42*. *apl-1* expression is upregulated during the last larval stage in hypodermal seam cells which is transcriptionally regulated by *hbl-1, lin-41 and lin-42*. Moreover, the level of the *apl-1* transcription is modulated by the activity of let-7 family of microRNAs.^1^ This work places apl-1 in a developmental timing pathway and may provide new insights into the time-dependent progression of AD. Kong et al reported down-regulation of let-7 in the adult AD-like brain using adult-onset *Drosophila* AD model.^2^ Kumar et al., reported a 7-miRNA signature including hsa-let-7g-5p that distinguished AD from controls with an AUC of 0.953.^3^ Chen et al., re-analyzed 10 miRNA datasets and identified hsa-let-7g-5p as down-regulated in at least 2 out of 10 datasets.^4^ For the related let-7 family members, let7a overexpression was shown to enhance the effect of neurotox­icity induced by Aβ_1‑40_ via regulation of autophagy, and the PI3K/Akt/mTOR signaling in a PC12 cell model of AD.^5^ Lehmann et al., further showed that extracellular let-7 activated the RNA-sensing Toll-like receptor 7 (*TLR7*) which played a role in innate immune response and induced neurodegeneration through neuronal *TLR7*. Introduction of CSF from individuals with AD containing increased amounts of let-7b into the CSF of wild-type mice resulted in neurodegeneration. Mice lacking TLR7 were resistant to this neurodegenerative effect, but this susceptibility to let-7 was restored in neurons transfected with *TLR7*.^6^ CSF from AD patients contained higher amounts of let-7b and let-7e miRNAs compared to healthy controls, while no differences were observed regarding the other seven members of let-7 miRNA family.^7^ In our study, hsa-let-7g-5p was up-regulated in STG (Tables 2 and 3).

Tau hypothesis

miR-34a was shown to repress the expression of endogenous tau protein in human neuroblastoma cell line M17D cells. Conversely, inhibition of endogenously expressed miR-34 family members leaded to increased endogenous tau expression.^8^ hsa-miR-34a-5p was identified as suggestively up-regulated (*p* = 1.89 x 10^-7^) in Takousi et al., 2019 meta-analysis^9^ and was also up regulated in this study (Tables 2 and 3).

Synaptic plasticity

Synapse degeneration is one of the underlying causes for progressive memory decline over the AD disease course. Recent observations suggested that the accumulation of the Wnt antagonist Dickkopf-1 (Dkk1) in the AD brain plays a critical role in triggering synaptic loss. Dkk1 cooperates with its transmembrane receptor Kremen1 (Krm1) to block the Wnt/β-catenin signaling pathway. miRNA-431 has shown to prevent Aβ-induced synapse loss in neuronal cell culture model of AD by silencing Krm1 (Ross et al., 2018). In this study, hsa-miR-431-3p was nominally down-regulated in the IFG (*p* = 0.03, Supplemental Table 1).

Immune-related signaling

miR-146a targets IL-1 receptor-associated kinase 1 (*IRAK1*) and TNF receptor-associated factor 6 (*TRAF6*) which are key adapter molecules in TLR and IL-1 receptor signaling cascades, mediates activation of NF-κB and AP-1 pathways.^10^ miR-146a also down-regulates complement factor H (CFH), an important repressor of the inflammatory response in the brain, highlighting the inflammatory component in the AD pathogenesis.^11^ hsa-miR-146a-5p was up-regulated in AD in the STG in this study (Table 2), consistent with the reported suggestively up-regulation (*p* = 4.88 x 10^-7^) in Takousi et al., 2019 meta-analysis^9^. Herrera-Espejo et al reported that hsa-miR-146a-5p was mostly up-regulated ^12^. Baseline miR-146a (*p* = 0.036) was recently showed to be up-regulated in patients with MCI who later converted to AD (i.e. correlated with disease progression).^13^ Higher CSF miR-146a level was also associated with AD hallmarks such as lower level of amyloid beta, presence of *APOE* ε4 allele, smaller volume of the hippocampus, CA1, and the subiculum subfields.^13^

hsa-miR-296-3p was identified as significantly associated with Braak neurofibrillary stage (*p* = 0.0001, FDR adjusted p-value = 0.01, Supplemental Table 5) in the IFG in this study. Several predicted targets of high confidence from Ingenuity Knowledge Base such as *CCR3*, *CD200R1*, *CX3CR1*, *NTF3*, *PPP3R2* were involved in neuroinflammation (Supplemental Table 3F). Both miR-146a and miR-296-3p were part of reliable or informative biomarkers differentially expressed in AD.^14^ miR-296 was also identified as differentially expressed in the oxidative stressed primary hippocampal neurons and hippocampus of senescence accelerated mice to capitulate the role of oxidative stress in the etiology and pathogenesis of AD.^15^

miRNAs with other corroborating evidence

Serum hsa-miR-501-3p levels were down-regulated in AD patients, and its lower levels significantly correlated with lower Mini-Mental State Examination scores. Contrary to its serum levels, hsa-miR-501-3p was remarkably up-regulated in the same donors’ AD brains.^16^ In this study, hsa-miR-501-3p was up-regulated in STG (Table 2), consistent with the reported strongly up-regulation (*p* = 2.03x10^-11^) in brain in the recent meta-analysis.^9^

While plasma exosomal miR-152-3p was also reported to be associated with AD,^17^ miR-152-5p that was nominally down-regulated in AD in both IFG (*p* = 0.001) and STG (*p* = 0.02, Supplemental Table 1) and also negatively correlated with Braak neurofibrillary stage in the IFG (*p* = 0.00006, adjusted p-value = 0.03, Supplemental Table S5). Down-regulation of miR-125b was also noted in serum and CSF of AD patients.^18^ hsa-miR-125b-2-3p was nominally up-regulated in the STG in this study (*p* = 0.009, Supplemental Table 1).

Potential Novel Finding

hsa-miR-592 (*p* = 0.009, adjusted p-value = 0.08) was suggestively associated with AD in the STG. In a mouse Crohn’s disease (CD) model, activation of G-protein-coupled estrogen receptor (GPER) was accompanied by reduction of inflammation (Jacenik et al., 2019). An agonist for GPCR, ICI 182.780, has been shown to be accompanied by overexpression of miR-592 (*p* < 0.001). Additionally, lower expression of miR-148-5p was documented after 17β-estradiol treatment (*p* < 0.05) (Jacenik et al., 2019). In our study, we observed a nominal down-regulation of miR-592 in the STG, and up-regulation of miR-148b-3p was observed in the STG (Supplemental Table 1 and Table 2), suggesting an increased inflammation state in AD compared to controls. miR-148b-3p and miR-592 levels were also negatively correlated (r = -0.33, *p* = 2.85 x 10^-5^, Supplemental Figure 3). Some of miR-148b-3p putative targets include major histocompatibility complex, class I, A (*HLA-A*); major histocompatibility complex, class I, B (*HLA-B*); and major histocompatibility complex, class I, C (*HLA-C*); SOS Ras/Rac guanine nucleotide exchange factor 1 (*SOS1, p* = 0.02 nominally down-regulated in the paired STG samples, implicated in immune regulation); SOS Ras/Rac guanine nucleotide exchange factor 2 (*SOS2*), Rho associated coiled-coil containing protein kinase 1 (*ROCK1*); and interleukin 1 receptor like 1 (*IL1RL1*) (Supplemental Tables 3A or 3C). Additional putative targets of miR-148b-3p such as roundabout guidance receptor 1 (*ROBO1, p* = 0.01 nominally down-regulated in the paired STG samples), reticulon 4 (*RTN4*), ADAM metallopeptidase with thrombospondin type 1 motif 5 (*ADAMTS5*), and neuropilin 1 (*NRP1*) suggest that miR-148b-3p may play a role in axonal guidance signaling/neurogenesis. Other putative targets of miR-148b-3p, such as cathepsin A (*CTSA*), tubulin epsilon 1 (*TUBE1*), VPS37A subunit of ESCRT-I (*VPS37A*), protein disulfide isomerase family A member 3 (*PDIA3*), protein kinase C zeta (*PRKCZ*), *HLA-A*, *HLA-B*, and *HLA-C* suggests that it might play a role in autophagy. It is worth to mention though that miR-148b-3p is not the only miRNA that correlated with miR-592, co-expression of miRNAs and miRNA-mRNA may imply that they are involved in similar biological process, which shall be validated by future experiments.

**Supplemental Figure 1** Association with Braak stage for hsa-miR212-5p in the IFG (A), STG (B), hsa-miR-132-5p in the in the IFG (C) and STG (D)


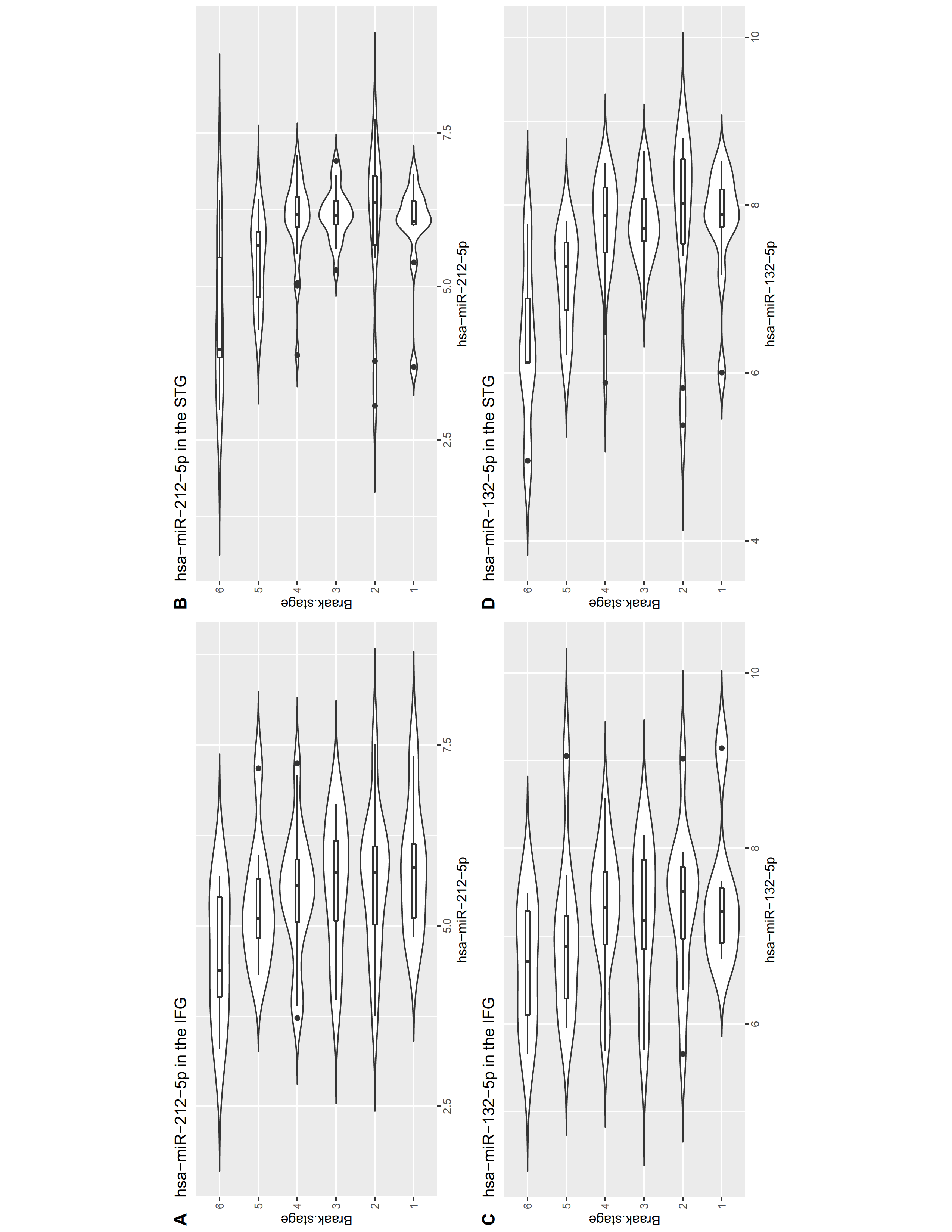


**Supplemental Figure 2** Negative correlation between hsa-miR-4446-3p and a panel of genes (A) *ZNF385A* (B) *UBE2Q1* (C) *USF2* and (D) *AUP1* color coded by clinical diagnosis (I) and subject (II). For the latter, the repeated measures correlation was calculated.

(I)


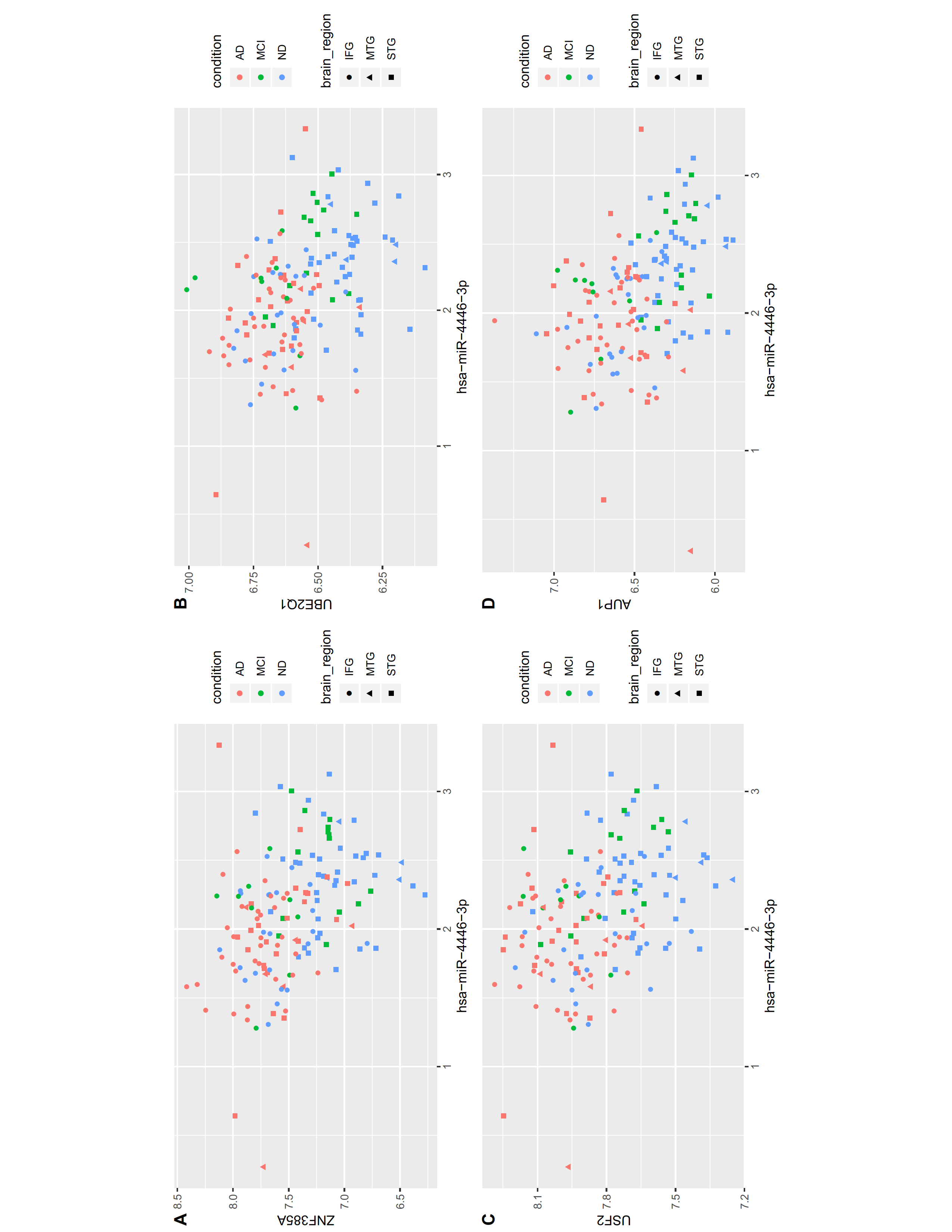


**(II)**

**
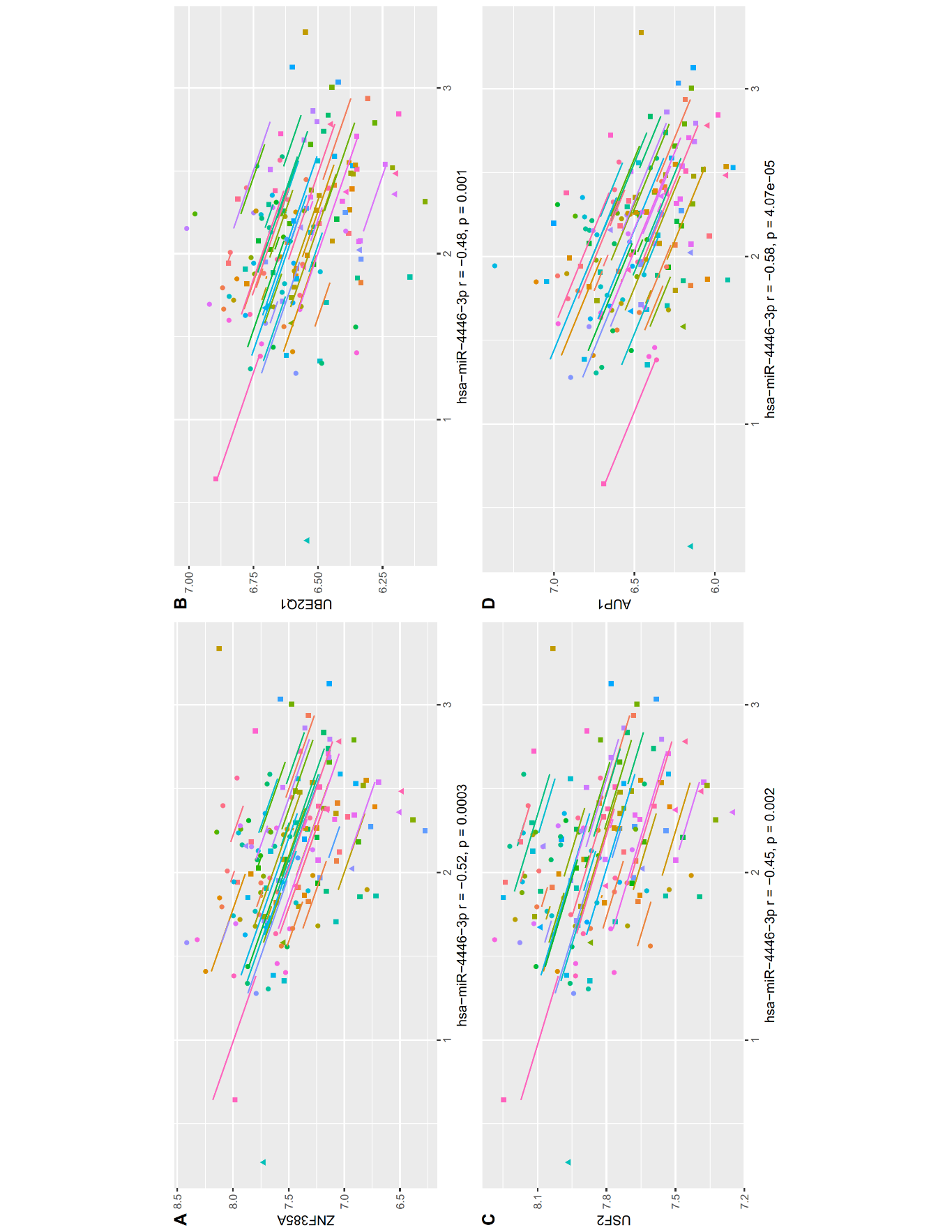
**

**Supplemental Figure 3** Anti-correlation between hsa-miR-592 and hsa-miR-148b-3p


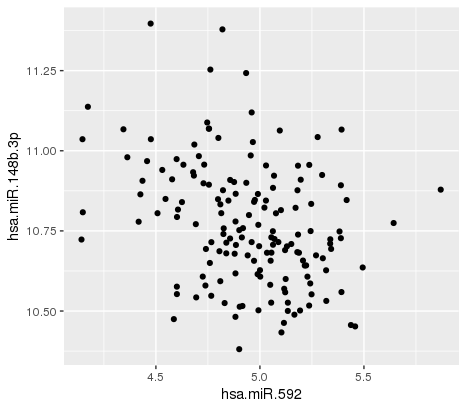


**Reference**

1. Niwa, R., Zhou, F., Li, C. & Slack, F.J. The expression of the Alzheimer's amyloid precursor protein-like gene is regulated by developmental timing microRNAs and their targets in Caenorhabditis elegans. *Dev Biol* **315**, 418-25 (2008).

2. Kong, Y., Wu, J. & Yuan, L. MicroRNA expression analysis of adult-onset Drosophila Alzheimer's disease model. *Curr Alzheimer Res* **11**, 882-91 (2014).

3. Kumar, P. *et al.* Circulating miRNA biomarkers for Alzheimer's disease. *PLoS One* **8**, e69807 (2013).

4. Chen, J. *et al.* MicroRNA expression data analysis to identify key miRNAs associated with Alzheimer's disease. *J Gene Med* **20**, e3014 (2018).

5. Gu, H., Li, L., Cui, C., Zhao, Z. & Song, G. Overexpression of let-7a increases neurotoxicity in a PC12 cell model of Alzheimer's disease via regulating autophagy. *Exp Ther Med* **14**, 3688-3698 (2017).

6. Lehmann, S.M. *et al.* An unconventional role for miRNA: let-7 activates Toll-like receptor 7 and causes neurodegeneration. *Nat Neurosci* **15**, 827-35 (2012).

7. Derkow, K. *et al.* Distinct expression of the neurotoxic microRNA family let-7 in the cerebrospinal fluid of patients with Alzheimer's disease. *PLoS One* **13**, e0200602 (2018).

8. Dickson, J.R., Kruse, C., Montagna, D.R., Finsen, B. & Wolfe, M.S. Alternative polyadenylation and miR-34 family members regulate tau expression. *J Neurochem* **127**, 739-49 (2013).

9. Takousis, P. *et al.* Differential expression of microRNAs in Alzheimer's disease brain, blood, and cerebrospinal fluid. *Alzheimers Dement* (2019).

10. Taganov, K.D., Boldin, M.P., Chang, K.J. & Baltimore, D. NF-kappaB-dependent induction of microRNA miR-146, an inhibitor targeted to signaling proteins of innate immune responses. *Proc Natl Acad Sci U S A* **103**, 12481-6 (2006).

11. Lukiw, W.J., Zhao, Y. & Cui, J.G. An NF-kappaB-sensitive micro RNA-146a-mediated inflammatory circuit in Alzheimer disease and in stressed human brain cells. *J Biol Chem* **283**, 31315-22 (2008).

12. Herrera-Espejo, S., Santos-Zorrozua, B., Alvarez-Gonzalez, P., Lopez-Lopez, E. & Garcia-Orad, A. A Systematic Review of MicroRNA Expression as Biomarker of Late-Onset Alzheimer's Disease. *Mol Neurobiol* (2019).

13. Ansari, A. *et al.* miR-146a and miR-181a are involved in the progression of mild cognitive impairment to Alzheimer's disease. *Neurobiol Aging* **82**, 102-109 (2019).

14. Denk, J. *et al.* MicroRNA Profiling of CSF Reveals Potential Biomarkers to Detect Alzheimer`s Disease. *PLoS One* **10**, e0126423 (2015).

15. Zhang, R. *et al.* Screening of microRNAs associated with Alzheimer's disease using oxidative stress cell model and different strains of senescence accelerated mice. *J Neurol Sci* **338**, 57-64 (2014).

16. Hara, N. *et al.* Serum microRNA miR-501-3p as a potential biomarker related to the progression of Alzheimer's disease. *Acta Neuropathol Commun* **5**, 10 (2017).

17. Lugli, G. *et al.* Plasma Exosomal miRNAs in Persons with and without Alzheimer Disease: Altered Expression and Prospects for Biomarkers. *PLoS One* **10**, e0139233 (2015).

18. Galimberti, D. *et al.* Circulating miRNAs as potential biomarkers in Alzheimer's disease. *J Alzheimers Dis* **42**, 1261-7 (2014).
